# Morphological Pathways of Mitochondrial Division

**DOI:** 10.3390/antiox7020030

**Published:** 2018-02-15

**Authors:** Bernard Tandler, Charles L. Hoppel, Jason A. Mears

**Affiliations:** 1 Center for Mitochondrial Disease, Cleveland, OH 44106, USA; bxt20@case.edu (B.T.); clh5@case.edu (C.L.H.); 2 Department of Biological Sciences, School of Dental Medicine, Case Western Reserve University, Cleveland, OH 44106, USA; 3 Department of Pharmacology, School of Medicine, Case Western Reserve University, Cleveland, OH 44106, USA; 4 Department of Medicine, School of Medicine, Case Western Reserve University, Cleveland, OH 44106, USA

**Keywords:** mitochondria, morphology, mitochondrial fission and fusion, inter-organelle contacts, mitochondrial dynamics

## Abstract

Mitochondrial fission is essential for distributing cellular energy throughout cells and for isolating damaged regions of the organelle that are targeted for degradation. Excessive fission is associated with the progression of cell death as well. Therefore, this multistep process is tightly regulated and several physiologic cues directly impact mitochondrial division. The double membrane structure of mitochondria complicates this process, and protein factors that drive membrane scission need to coordinate the separation of both the outer and inner mitochondrial membranes. In this review, we discuss studies that characterize distinct morphological changes associated with mitochondrial division. Specifically, coordinated partitioning and pinching of mitochondria have been identified as alternative mechanisms associated with fission. Additionally, we highlight the major protein constituents that drive mitochondrial fission and the role of connections with the endoplasmic reticulum in establishing sites of membrane division. Collectively, we review decades of research that worked to define the molecular framework of mitochondrial fission. Ongoing studies will continue to sort through the complex network of interactions that drive this critical event.

Omne granulum e granulo

Altmann, 1890 [1]

## 1. Early Recognition of Mitochondrial Division 

### 1.1. Mitochondrial Partitioning Is Observed

That mitochondria are the powerhouses of the cell [2] has assumed the proportions of a cliché. These organelles are universally acknowledged to be the principal progenitors of ATP. Even before their function(s) had been established, an intriguing puzzle came to the fore. It was obvious that cells maintained numerically uniform populations of mitochondria as well as a uniform mitochondrial mass in the face of multiple cell divisions. What was the origin of these newborn organelles? Three ideas, based entirely on light microscopy, gained currency: (1) they were generated by other cell constituents; (2) they arose de novo; (3) they were self-replicating, i.e., they could divide (reviewed by Novikoff [3] and by Lehninger [4]). With the discovery of mitochondrial DNA [5,6], the idea that mitochondria were capable of division gained ascendency.

**Figure 1:**
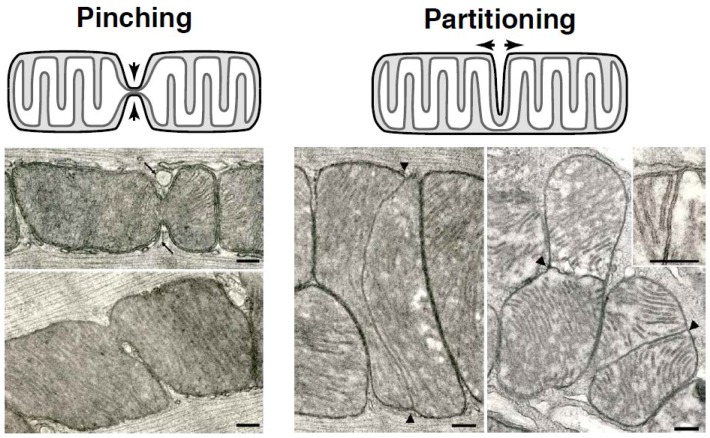
In skeletal muscle, mitochondrial division proceeds through alternative mechanisms termed pinching and partitioning. For pinching (left side), the outer membranes on opposite sides of a mitochondrion make deep invaginations at sites of ensuing fission. Adjacent sarcoplasmic reticulum membranes are highlighted (arrows). For partitioning (right side), elongated cristae span the width of the organelle and maintain direct continuity with the boundary membrane at either end. Subsequent in-growth of the outer membranes would then lead to mitochondrial fission. Scale bar = 0.2 µm.

Ingenious biochemical and autoradiographic studies [7,8,9] provided convincing evidence that mitochondria do indeed divide, at least in lower organisms. However, the morphological parameters in this process remained questionable. A number of studies reported the presence of mitochondria in a variety of mammalian cell types that were characterized by a partition, usually medially placed (see compendium by Tandler et al., 1969 [10]). Partitions have the lineaments of an elongated crista that spans the width of the organelle and is in direct continuity with the boundary membrane at either end (Figure 1). Such mitochondrial configurations usually were described as indicative of mitochondrial fission, but all descriptions included the caveat that they might represent a stage in mitochondrial fusion. The directionality of the process was finally settled by observations of the steps in the normalization of giant mitochondria (megamitochondria) [10]. Outsized mitochondria could be produced in mouse hepatocytes simply by placing test animals on a riboflavin (vitamin B_2_)-free diet [11]. After six weeks, some mitochondria in virtually every hepatocyte had attained dimensions as great or greater than the cell nuclei. Aside from their size, the giant mitochondria were virtual simulacra of their normal counterparts. They had the same number and arrangement of cristae per unit area of mitochondrial matrix. This size increase was occasioned by both growth and fusion of mitochondria—the latter process was accompanied by a significant reduction in the number of mitochondria per cell. When these mice were restored to a normal, vitamin B_2_-replete diet, the mitochondria returned to normal size and numbers by the end of three days. At 24 hours of recovery, examination of hepatocytes revealed a large number of partitioned megamitochondria. Most of the partitions were at the base of small surface buds, which usually matched normal mitochondria in size. Ingrowth of the outer membrane into the partition ultimately led to organelle scission, and continued repetition of this process resulted in a population of hepatic mitochondria of typical size. Clearly, partitioned mitochondria were dividing—if they had been fusing, they would have gotten bigger, rather than smaller.

When mice were fed galactoflavin, a competitive inhibitor of riboflavin, their hepatic mitochondria attained megamitochondria size in approximately three weeks, but their recovery—once the animals were restored to a normal diet—was relatively prolonged, a matter of 10–14 days [12]. During this interval, partitioned organelles were quite common. Clearly, it required a longer time for mitochondrial recovery because of the persistence of the galactoflavin.

Of course, the foregoing studies dealt with experimentally altered cells. The question as to whether the same process occurs under physiological conditions was answered by the observations of Larsen [13], who studied the fat body of the skipper butterfly, *Calpodes ethlius*. In this insect, the fat body (analogous to the mammalian liver) undergoes a wave of autophagy near the end of the penultimate stage of development that results in the almost total effacement of the resident mitochondrial population. Shortly after emergence of the adult, there is an explosion of mitochondrial replication. During this mitochondrial restoration, partitioned mitochondria are everywhere in the fat body. Some of these organelles display more than one partition. The final result is an increase of ~700% in the number of normal size organelles. The conclusion was unavoidable—mitochondria divide by partition formation; this determination applies not only to megamitochondria, but to normal-sized organelles in a host of different tissues as well (see compendium by Tandler et al. [10]).

The genesis of partitions is of some interest. The simplest mechanism for this origin is that an individual crista is stimulated to elongate until it spans its host mitochondrion and contacts the antipodal boundary membranes, with which it ultimately fuses. It has now formed a roadway for the ingrowth of the outer membrane, which finally leads to organelle division. Fawcett [14] presents a gallery of micrographs of the stages in partition formation in conventional hepatic mitochondria from the earliest partitions through the seriatim intrusion of the outer membrane into this structure. That the story may be more complex is illustrated by an electron micrograph taken by Daniel Friend and featured in Fawcett’s ultrastructural atlas. This micrograph depicts a dividing adrenocortical mitochondrion. Such organelles have tubular cristae as shown by scanning electron microscopy, but when these mitochondria are thin-sectioned for transmission electron microscopy, the transected cristae appear to have a vesicular form, with the result that mitochondria in steroid-secreting cells are usually described as vesicle-containing. In the organelle in question, the matrix is replete with “vesicles”, but a partition bisects the mitochondrion into two contiguous compartments, the exemplar of division. The morphological difference between the lamelliform partition and the tubular cristae suggests that these two structures may have differing molecular composition and, by extension, different biochemical functions. This contrasting configuration implies that there are specific domains on the boundary membrane that respond to the signal for organelle replication by generating a structure that varies from its crista companions and is directed solely towards mitochondrial division.

Ultimately, an impressive number of studies appeared that illustrated the production of giant mitochondria, usually in liver cells, by pharmacological and nutritional manipulation (reviewed by Tandler and Hoppel, 1986 [15]). Of these, the copper-chelating agent, cuprizone (biscyclohexanone oxaldihydrazone), was of particular interest. In mice fed cuprizone for just a few days, there was an obvious increase in the number and mass of cardiac mitochondria. Partitioned mitochondria were present at this juncture in significant numbers [16]. Although cardiac mitochondria of this configuration do in fact occur in normal, untreated hearts, they are quite rare and difficult to detect [17]. In the cuprizone-treated hearts, the partitioned mitochondria generally were of typical size but displayed an unusual disposition of their cristae. Cristae on opposite sides of the partition were in an orthogonal arrangement, that is, if one set was seen in profile, its opposite number was seen en face. Biochemical studies of cuprizone-treated hearts subsequently confirmed the increase in mitochondrial mass originally noted by electron microscopy [18].

### 1.2. Mitochondrial Pinching—A Distinct Fission Pathway

As far as the capacity for the induction of megamitochondria by cuprizone is concerned, addition of this agent to the diet of mice rapidly results in the formation of giant hepatic mitochondria as well [19]. These outsize organelles are morphologically different from those engendered by ariboflavinosis: their short, relatively sparse, evenly dispersed cristae extend inward in the manner of a picket fence, while their matrix compartment is essentially free of membranes. Return of these megamitochondria to a normal size occurred in an astonishingly short time and was occasioned by the simple expedient of omitting the curprizone from the diet [20]. A few hours after the mice resumed ingesting their conventional food, the hitherto globular megamitochondria became pleomorphic. Many underwent medial attenuation. The continued narrowing of these organelles finally led to their division. Continued repetition of this process quickly resulted in complete normalization in morphological terms of the hepatic mitochondria. This process came to be known under different circumstances as “pinching” (Figure 1).

The matrices of all mitochondria contain numerous molecules of mitochondrial DNA (mDNA), each of which is secured to the boundary membrane. Irrespective of pathway, when mitochondria divide—either by partition formation or by pinching—a portion of the boundary membrane, together with its attached retinue of mDNA, perforce ends up in each of the daughter organelles, even when these progeny are of greatly unequal volume. This being the case, there generally is sufficient mDNA present in the newly generated mitochondria, independent of size, to allow these cytological offspring to carry out their canonical functions.

It is clear that partitioning and pinching of mitochondria involve either lengthening or shrinkage of the cristae and outer membrane, respectively. Because of the surface area to volume relationships, new membrane must be added to mitochondria during partitioning. During this process, one or more of the cristae lengthen to a point where it spans the inner compartment. Subsequent in-growth of the outer mitochondrial membrane continues until it reaches the inner membrane, to which it joins. The four-layered partition thus formed marks the dividing line between sibling organelles. In pinching mitochondria, cytosolic proteins and adjacent organelles are positioned to constrict mitochondria, and the impinging outer membrane topologically restricts the inner membrane. This contraction prunes the mitochondria into smaller compartments.

What controls the selection mode of mitochondrial division is not known. In some cells, the location of the mitochondria seems to play a cardinal role. For example, in the rat heart, partitioned mitochondria are found solely among subsarcolemmal mitochondria, whereas pinching mitochondria predominate among interfibrillar organelles. In contrast, the exact opposite locations are characteristic of heart mitochondria in mice. In skeletal muscle, both types of mitochondrial division take place in all venues of the muscle fibers. Selection of the mode of mitochondrial fission is due to an as yet uncharacterized mechanism.

### 1.3. Alternative Fission Pathways

Partition formation and pinching are by no means the only modalities of mitochondrial division. In many insects, spermatocyte mitochondria have the form of elongated rods. In anaphase, these organelles surround the middle of the spindle like the staves of a picket fence [21]. Wilson [22] states that there is no doubt that many of these mitochondria are cut across their equator during cell division (presumably by the constricting cleavage furrow).

An ordered and symmetrical sequence of mitochondrial division takes place in hemipteral spermatocytes of the scorpion, *Centrurus* [23]. Here, the mitochondria are fused into a single, ring-shaped body, the nebenkern, which assumes a tangential position alongside the spindle; it is transversely cut into two half-rings by cell division. Each resulting hipposiderous nebenkern undergoes a series of divisions so that each spermatid receives two segments of the original toroid body. Because of the limited resolution of the light microscopes used in these studies, it was not possible to trace the precise details of nebenkern division in these insects, but it seems likely that plasma membranes, rather than intrinsic mitochondrial membranes, are the tools whereby mitochondria are bisected to retain constancy of mitochondrial number and ultimately of mass.

After these studies appeared, investigation of mitochondrial division languished for some time. The study of mitochondrial dynamics in living (cultured) cells signaled a revival of interest in the biography of these organelles. Phase contrast microscopy was used to confirm the many observations based on conventional microscopy of mitochondrial motility and shape changes in cultured cells (see compendium by Bereiter Hahn, [24]). Ultimately, study of these cells by fluorescence microscopy, and especially by confocal microscopy, led to the notion that mitochondria divide and fuse with one another in a never-ending dance.

## 2. The Molecular Ensemble Driving Mitochondrial Division

### 2.1. The Identification of Protein Machineries

**Figure 2:**
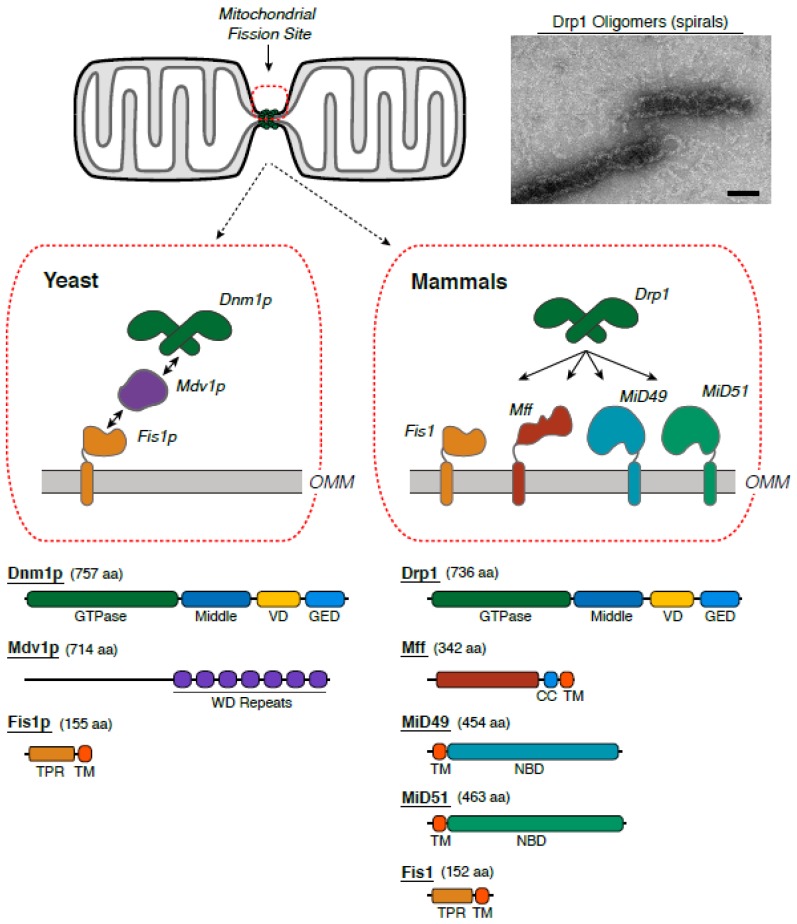
The molecular components of the mitochondrial fission machinery are presented. In yeast, mitochondrial fission is mediated by Dnm1p. Fis1p is anchored in the outer mitochondrial membrane (OMM), and Mdv1 is an adaptor for assembly of the multicomponent fission machinery. In mammals, Drp1 drives membrane fission. When a non-hydrolyzable guanosine-5’-triphosphate (GTP) analog (βγ-Methyleneguanosine-5′-triphosphate, or GMPPCP) is added to Drp1, it spontaneously forms oligomers that represent the contractile core of the fission apparatus. Several receptor proteins have been identified, including Fis1, Mff, MiD49, and MiD51. The primary sequence and protein domains are highlighted. Abbreviations: VD, variable domain; GED, GTPase effector domain; CC, coiled-coil domain; TM, transmembrane domain; NBD, nucleotide-binding domain; TPR, tetratricopeptide repeat; WD: repeating units containing a conserved core of approximately 40 amino acids that usually end with a tryptophan-aspartic acid dipeptide.

Investigation of mitochondrial division quickly moved into the molecular realm, with yeast cell mitochondria gaining primacy as the objects of study. A dynamin-related protein, Dnm1p, was found to be the master regulator of mitochondrial fission [25,26]. Proteins in the dynamin family regulate membrane fission and fusion events throughout the eukaryotic cell [27,28]. To achieve this task, these proteins self-assemble into large polymers capable of imparting force at defined membrane sites (Figure 2). Concordantly, fluorescence studies identified the formation of defined Dnm1p puncta at sites of ensuing mitochondrial fission [29]; subsequent electron microscopy (EM) analysis of mitochondrial constriction sites found Dnm1p forming spirals around “pinched” membranes [30]. Like other dynamin-related proteins, Dnm1p assembled into large helical polymers powered by guanosine-5’-triphosphate (GTP) hydrolysis to actively constrict membranes in vitro [31,32].

Unlike dynamin, which mediates vesicle release at various cellular membranes, Dnm1p did not contain a canonical lipid-binding domain to drive membrane interactions. Rather, partner proteins were identified as necessary constituents of multi-component mitochondrial fission machineries. Recruitment of Dnm1p from the cytosol to the surface of mitochondrial was mediated by mitochondrial fission 1 protein (Fis1p) and mitochondrial division protein 1 (Mdv1) [33,34]. Fis1 is a tail-anchored protein in the outer mitochondrial membrane (OMM). Mdv1 was shown to be an adaptor protein believed to bridge Dnm1-Fis1 interactions [35]. Based on these findings, a central dogma prevailed wherein partner proteins coordinated targeting of Dnm1p to mitochondrial fission sites (Figure 2). However, Mdv1p was shown to directly regulate Dnm1p activity [36], which indicated that these receptors were more than mere scaffolds.

Naturally, comparable studies were pursued to identify similar proteins in the mammalian mitochondrial fission machinery. Dynamin-related protein 1 (Drp1), the Dnm1p homolog, was found to serve an essential role in mammalian mitochondrial division [37,38]. While a Fis1p homolog was also discovered [39,40], no Mdv1p homolog was present in higher eukaryotes. Rather, new partner proteins were shown to contribute to Drp1 targeting and membrane fission. Specifically, mitochondrial fission factor (Mff) was discovered as a distinct tail-anchored membrane protein that recruits Drp1 to the OMM [41,42]. Separate studies found additional integral membrane mitochondrial division proteins (MiD49 and MiD51) that co-localize with Drp1 and regulate mitochondrial dynamics [43,44]. So, while similarities exist in yeast and mammals, significant differences are apparent (Figure 2). Specifically, the combinatorial complexity of potential Drp1 interactions in mammals raises the question of whether a singular fission process exists. Based on the morphologically distinct processes of pinching and partitioning described previously (Figure 1), it seems plausible that separate protein interactions could facilitate distinct membrane remodeling events. At the same time, coordinated interactions between Drp1 and its partners likely offer combinatorial resources for regulating mitochondrial division, which serves several critical functions related to both cell proliferation [45,46,47,48,49] and cell death [50,51,52,53,54]. More specifically, this conserved membrane fission event is essential for mitochondrial inheritance, quality control via sequestration of damaged regions of the organelle, termed mitophagy, transport of mitochondria to the periphery of cells, and the intrinsic apoptotic cascade, which involves the release of cytochrome *c*. The specific protein interactions that allow Drp1 to govern these disparate processes are largely unknown. The wide-ranging network of interactions provides one explanation for how the mitochondrial fission machinery can be modulated.

### 2.2. Assorted Factors Regulate the Fission Machinery

**Figure 3:**
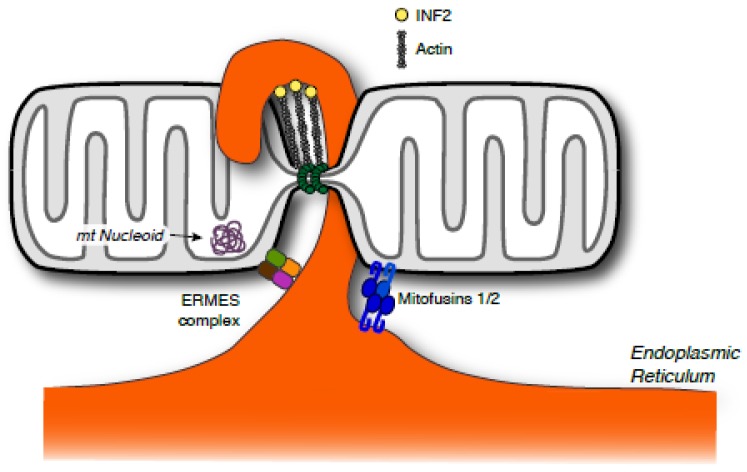
Mitochondrial interactions with the endoplasmic reticulum mark sites of ensuing fission. This connection is mediated by different protein tethering complexes, including mitofusin heteropolymers and the endoplasmic reticulum-mitochondria encounter structure (ERMES) complex. Proteins on the endoplasmic reticulum (ER) membrane serve as nucleation sites for building actin filaments that also constrict mitochondria and may provide tension needed during the membrane scission event. INF2: Inverted formin 2.

Cellular signaling cues also have been identified that directly impact Drp1 and its partners to regulate the efficiency of mitochondrial membrane remodeling. Specifically, mitochondrial fission proteins are “tuned” by post-translational modifications (PTMs) to modulate interactions within the fission machinery in response to calcium flux [55,56,57,58], cell cycle progression [46,49,59], oxidative stress [60,61,62] and cellular energy levels [63,64]. These sequence changes have been shown to alter the Drp1 localization and activity in cells, which directly enhances or impedes mitochondrial membrane remodeling. Within Drp1, PTMs are most abundant in a sequence region called the variable domain (VD, Figure 3). Interestingly, the VD is known to regulate Drp1 self-assembly [65] and serves as a lipid-binding domain [66,67]. More recently, this region also was shown to suppress interactions with the partner protein, Mff [68]. Therefore, PTMs in this region of Drp1 likely augment or inhibit critical intra- and inter-molecular interactions to regulate the efficiencies of assembly, constriction, and subsequent release of the fission complex at the mitochondrial membrane surface.

Drp1 interactions with specific lipid species in the OMM also are tightly regulated [69], and the exposure of the mitochondrial-specific lipid, cardiolipin, has been shown to potently stimulate Drp1 activity [66,67,70]. In reconstitution experiments, interactions with negatively charged lipid templates promote Drp1 self-assembly to form large helical oligomers [65,71,72,73,74]. Similarly, addition of non-hydrolyzable GTP analogs induces polymerization of Drp1 into large oligomers (Figure 2), and these assemblies represent the core of the mitochondrial fission machinery. In this way, in vitro studies can be used to mimic the interactions that occur at membrane sites in vivo, and the impact of specific interactions can be assessed. In the case of lipid interactions, Drp1 and Dnm1p have been shown to induce the formation of oligomers that exhibit a broad range of diameters, which may reflect the inherent ability of these proteins to accommodate mitochondrial membranes with different morphologies. Interestingly, sequence changes in the aforementioned VD also modulate the geometry of Drp1 polymers formed in the presence of lipid templates [75]. Structural studies are currently being pursued to define the critical interactions within Drp1 oligomers that drive the assembly of these large protein complexes. Interactions with specific lipids have been shown to affect the architecture of Drp1 assembly and thereby regulate the fission machinery [76]. Partner proteins, such as Mff, MiD49/51, and Fis1, also will interact with Drp1 and have the potential to impact its self-assembly properties. Critically, the spatiotemporal relationship of when and where these interactions occur is central to understanding how mitochondrial fission proceeds. Clearly, membrane microenvironments dictate recruitment and processivity of the fission machinery.

### 2.3. Inter-Organelle Connections Come to the Fore

Because interactions between mitochondria and the endoplasmic reticulum (ER) are important for the transfer of phospholipids and calcium [77,78,79,80], connections with neighboring organelles point to sites of ensuing mitochondrial fission [81,82]. Previous EM analyses found that the ER and mitochondria were closely juxtaposed (arrows, Figure 1), which provides the opportunity for more efficient communication and exchange of materials between these organelles. This interaction is preserved in yeast and mammals; however, the protein constituents that tether them appear to be distinct, much like the fission machinery. In yeast, an ER-mitochondria encounter structure (ERMES, Figure 3) is found at the interface between mitochondria and the ER [83,84]. This complex is implicated in the distribution of mitochondria and the exchange of lipids [85]. Interestingly, ERMES complexes are localized adjacent to mitochondrial DNA nucleoids and actively facilitate the segregation of mitochondrial DNA in coordination with the mitochondrial fission machinery [86].

In mammals, the ER membrane is tethered to mitochondria through distinct dynamin-related proteins, called mitofusins 1 and 2 (Mfn1 and Mfn2, Figure 3). These proteins are required for mitochondrial fusion, but this mitofusin-mediated connection is critical for mitochondrial calcium uptake from the ER [77]. Mitofusins are key mediators of mitochondrial fusion, so it is unclear whether these proteins can also coordinate membrane fission at these contact sites. Nevertheless, ER encirclement of mitochondria narrows the OMM before the fission ensues. This interaction has been shown to activate inverted formin 2 (INF2), a vertebrate formin protein that promotes actin polymerization [87]. More specifically, INF2 activity has been suggested to drive actin polymerization at ER-mitochondria contact sites to promote narrowing of the mitochondrial membrane and enhance assembly of Drp1 and other components of the fission machinery [82]. Subsequent Drp1 constriction would then resolve the fission event at membrane sites defined by ER interactions.

More recently, it has been suggested that dynamin 2, which plays an important role in the release of vesicles from biological membranes, i.e., endocytosis, is involved in mitochondrial fission [88]. The underlying mechanism for this event remains unknown, though dynamin likely enhances the efficiency of this complex membrane-remodeling event. Interestingly, this endocytic protein is not conserved in yeast, so it is unclear whether dynamin is an essential protein in mitochondrial division. Structural studies with the yeast Dnm1p identified a large helical geometry that could limit membrane constriction [31,32], but mitochondrial fission proceeds. Similar studies with mammalian Drp1 identified a narrower helical topology that can constrict mitochondrial membranes to diameters similar to those observed for dynamin–lipid tubules [65,67]. Still, the Drp1–membrane interaction may be more tenuous than dynamin–membrane interactions given the lack of a strong lipid-binding motif. Therefore, dynamin may enhance membrane scission by stabilizing connections at sites of ensuing fission. Additional studies are needed to discern the molecular interactions that facilitate dynamin 2 localization at sites of mitochondrial fission and how these interactions contribute to membrane remodeling.

## 3. Conclusions

Moving forward, the main goal for the research community will be to sort through the complex network of protein and lipid interactions that occur at sites of mitochondrial fission. Inter-organelle connections provide a platform for the constant exchange of interactions and signals that regulate the morphology of mitochondria, and thereby influence cell bioenergetics and viability. Moreover, the heterogeneity of these interactions may dictate the subsequent outcome of membrane remodeling events. Partition formation and pinching appear to be two distinct and separate modalities in mitochondrial division, but the causal interactions that dictate these membrane transformations are unknown. Given the diversity of cellular cues leading to mitochondrial fission, i.e., cell cycle progression, cell stress, mitophagy, etc., it is entirely possible that mitochondrial fission may be triggered through distinct interactions leading to diverse changes in organelle morphology and subsequent function.

For the better part of the past century, it has become clear that mitochondria respond to physiologic cues to alter intra- and inter-organelle connectivity, bioenergetic capacity, and permeability. We know that mitochondrial division is an essential process for life, but how does this process become corrupted to facilitate death during apoptosis? We know that fission partitions mitochondrial DNA to preserve organelle function, but it also partitions damaged regions of mitochondria targeted for degradation via mitophagy. The molecular underpinnings that compel these distinct events will provide new insight into the interactions that govern mitochondrial fission and how these interactions become altered to promote synergistic changes with cellular cues in mitochondrial function.

## References

[1] Altmann R. (1890). Die Elementarorganismen Und Ihre Beziehungen Zu Den Zellen.

[2] Siekevitz P. (1957). Powerhouse of the Cell. Sci. Am..

[3] Novikoff A.B. (1961). Mitochondria (Chondriosomes). Cell.

[4] Lehninger A.L. (1964). The Mitochondrion. Molecular Basis of Structure and Function.

[5] Nass M.M., Nass S. (1963). Intramitochondrial Fibers with DNA Charachteristics. I. Fixation and Electron Staining Reactions. J. Cell Biol..

[6] Rabinowitz M., Sinclair J., Desalle L., Haselkorn R., Swift H.H. (1965). Isolation of Deoxyribonucleic Acid from Mitochondria of Chick Embryo Heart and Liver. Proc. Natl. Acad. Sci. USA.

[7] Luck D.F. (1965). Formation of Mitochondria in *Neurospora Crassa*. A Study Based on Mitochondrial Density Changes. J. Cell Biol..

[8] Luck D.J. (1963). Formation of Mitochondria in *Neurospora Crassa*. A Quantitative Radioautographic Study. J. Cell Biol..

[9] Parsons J.A., Rustad R.C. (1968). The Distribution of DNA among Dividing Mitochondria of *Tetrahymena Pyriformis*. J. Cell Biol..

[10] Tandler B., Erlandson R.A., Smith A.L., Wynder E.L. (1969). Riboflavin and Mouse Hepatic Cell Structure and Function. II. Division of Mitochondria during Recovery from Simple Deficiency. J. Cell Biol..

[11] Tandler B., Erlandson R.A., Wynder E.L. (1968). Riboflavin and Mouse Hepatic Cell Structure and Function. I. Ultrastructural Alterations in Simple Deficiency. Am. J. Pathol..

[12] Tandler B., Hoppel C.L. (1974). Ultrastructural Effects of Dietary Galactoflavin on Mouse Hepatocytes. Exp. Mol. Pathol..

[13] Larsen W.J. (1970). Genesis of Mitochondria in Insect Fat Body. J. Cell Biol..

[14] Fawcett D.W. (1981). The Cell.

[15] Tandler B., Hoppel C.L. (1986). Studies on Giant Mitochondria. Ann. N. Y. Acad. Sci..

[16] Tandler B., Hoppel C.L. (1972). Possible Division of Cardiac Mitochondria. Anat. Rec..

[17] Fujioka H., Tandler B., Hoppel C.L. (2012). Mitochondrial Division in Rat Cardiomyocytes: An Electron Microscope Study. Anat. Rec..

[18] Medeiros D.M., Jennings D. (2002). Role of Copper in Mitochondrial Biogenesis via Interaction with ATP Synthase and Cytochrome C Oxidase. J. Bioenerg. Biomembr..

[19] Suzuki K. (1969). Giant Hepatic Mitochondria: Production in Mice Fed with Cuprizone. Science.

[20] Tandler B., Hoppel C.L. (1973). Division of Giant Mitochondria during Recovery from Cuprizone Intoxication. J. Cell Biol..

[21] Bowen R.H. (1920). Studies on Insect Spermatogenesis: I. The History of the Cytoplasmic Components of the Sperm in Hemiptera. Biol. Bull..

[22] Wilson E.B. (1925). The Cell in Heredity and Development.

[23] Wilson E.B. (1916). The Distribution of the Chondriosomes to the Spermatozoa in Scorpions. Proc. Natl. Acad. Sci. USA.

[24] Bereiter-Hahn J. (1978). Intracellular Motility of Mitochondria: Role of the Inner Compartment in Migration and Shape Changes of Mitochondria in XTH-Cells. J. Cell Sci..

[25] Otsuga D., Keegan B.R., Brisch E., Thatcher J.W., Hermann G.J., Bleazard W., Shaw J.M. (1998). The Dynamin-Related GTPase, Dnm1p, Controls Mitochondrial Morphology in Yeast. J. Cell Biol..

[26] Sesaki H., Jensen R.E. (1999). Division Versus Fusion: Dnm1p and Fzo1p Antagonistically Regulate Mitochondrial Shape. J. Cell Biol..

[27] Heymann J.A., Hinshaw J.E. (2009). Dynamins at a Glance. J. Cell Sci..

[28] Praefcke G.J., Mcmahon H.T. (2004). The Dynamin Superfamily: Universal Membrane Tubulation and Fission Molecules?. Nat. Rev. Mol. Cell Biol..

[29] Legesse-Miller A., Massol R.H., Kirchhausen T. (2003). Constriction and Dnm1p Recruitment are Distinct Processes in Mitochondrial Fission. Mol. Biol. Cell.

[30] Bleazard W., Mccaffery J.M., King E.J., Bale S., Mozdy A., Tieu Q., Nunnari J., Shaw J.M. (1999). The Dynamin-Related GTPase Dnm1 Regulates Mitochondrial Fission in Yeast. Nat. Cell Biol..

[31] Ingerman E., Perkins E.M., Marino M., Mears J.A., Mccaffery J.M., Hinshaw J.E., Nunnari J. (2005). Dnm1 Forms Spirals That are Structurally Tailored to Fit Mitochondria. J. Cell Biol..

[32] Mears J.A., Lackner L.L., Fang S., Ingerman E., Nunnari J., Hinshaw J.E. (2011). Conformational Changes in Dnm1 Support a Contractile Mechanism for Mitochondrial Fission. Nat. Struct. Mol. Biol..

[33] Mozdy A.D., Mccaffery J.M., Shaw J.M. (2000). Dnm1p Gtpase-Mediated Mitochondrial Fission is a Multi-Step Process Requiring the Novel Integral Membrane Component Fis1p. J. Cell Biol..

[34] Tieu Q., Okreglak V., Naylor K., Nunnari J. (2002). The WD Repeat Protein, Mdv1p, Functions as a Molecular Adaptor by Interacting with Dnm1p and Fis1p during Mitochondrial Fission. J. Cell Biol..

[35] Naylor K., Ingerman E., Okreglak V., Marino M., Hinshaw J.E., Nunnari J. (2006). Mdv1 Interacts with Assembled Dnm1 to Promote Mitochondrial Division. J. Biol Chem..

[36] Lackner L.L., Horner J.S., Nunnari J. (2009). Mechanistic Analysis of a Dynamin Effector. Science.

[37] Smirnova E., Shurland D.L., Ryazantsev S.N., Van Der Bliek A.M. (1998). A Human Dynamin-Related Protein Controls the Distribution of Mitochondria. J. Cell Biol..

[38] Smirnova E., Griparic L., Shurland D.L., Van Der Bliek A.M. (2001). Dynamin-Related Protein Drp1 is Required for Mitochondrial Division in Mammalian Cells. Mol. Biol. Cell.

[39] James D.I., Parone P.A., Mattenberger Y., Martinou J.C. (2003). hFis1, a Novel Component of the Mammalian Mitochondrial Fission Machinery. J. Biol. Chem..

[40] Yoon Y., Krueger E.W., Oswald B.J., Mcniven M.A. (2003). The Mitochondrial Protein hFis1 Regulates Mitochondrial Fission in Mammalian Cells through an Interaction with the Dynamin-Like Protein DLP1. Mol. Cell Biol..

[41] Gandre-Babbe S., Van Der Bliek A.M. (2008). The Novel Tail-Anchored Membrane Protein Mff Controls Mitochondrial and Peroxisomal Fission in Mammalian Cells. Mol. Biol. Cell.

[42] Otera H., Wang C., Cleland M.M., Setoguchi K., Yokota S., Youle R.J., Mihara K. (2010). Mff is an Essential Factor for Mitochondrial Recruitment of Drp1 during Mitochondrial Fission in Mammalian Cells. J. Cell Biol..

[43] Palmer C.S., Osellame L.D., Laine D., Koutsopoulos O.S., Frazier A.E., Ryan M.T. (2011). MiD49 and MiD51, New Components of the Mitochondrial Fission Machinery. EMBO Rep..

[44] Zhao J., Liu T., Jin S., Wang X., Qu M., Uhlen P., Tomilin N., Shupliakov O., Lendahl U., Nister M. (2011). Human MIEF1 Recruits Drp1 to Mitochondrial Outer Membranes and Promotes Mitochondrial Fusion Rather than Fission. EMBO J..

[45] Kashatus J.A., Nascimento A., Myers L.J., Sher A., Byrne F.L., Hoehn K.L., Counter C.M., Kashatus D.F. (2015). Erk2 Phosphorylation of Drp1 Promotes Mitochondrial Fission and MAPK-Driven Tumor Growth. Mol. Cell.

[46] Marsboom G., Toth P.T., Ryan J.J., Hong Z., Wu X., Fang Y.H., Thenappan T., Piao L., Zhang H.J., Pogoriler J. (2012). Dynamin-Related Protein 1-Mediated Mitochondrial Mitotic Fission Permits Hyperproliferation of Vascular Smooth Muscle Cells and Offers a Novel Therapeutic Target in Pulmonary Hypertension. Circ. Res..

[47] Mitra K., Wunder C., Roysam B., Lin G., Lippincott-Schwartz J. (2009). A Hyperfused Mitochondrial State Achieved at G1-S Regulates Cyclin E Buildup and Entry into S Phase. Proc. Natl. Acad. Sci. USA.

[48] Sastre-Serra J., Nadal-Serrano M., Pons D.G., Roca P., Oliver J. (2012). Mitochondrial Dynamics is Affected by 17beta-Estradiol in the MCF-7 Breast Cancer Cell Line. Effects on Fusion and Fission Related Genes. Int. J. Biochem. Cell Biol..

[49] Xie Q., Wu Q., Horbinski C.M., Flavahan W.A., Yang K., Zhou W., Dombrowski S.M., Huang Z., Fang X., Shi Y. (2015). Mitochondrial Control by DRP1 in Brain Tumor Initiating Cells. Nat. Neurosci..

[50] Frank S., Gaume B., Bergmann-Leitner E.S., Leitner W.W., Robert E.G., Catez F., Smith C.L., Youle R.J. (2001). The Role of Dynamin-Related Protein 1, a Mediator of Mitochondrial Fission, in Apoptosis. Dev. Cell.

[51] Fannjiang Y., Cheng W.C., Lee S.J., Qi B., Pevsner J., Mccaffery J.M., Hill R.B., Basanez G., Hardwick J.M. (2004). Mitochondrial Fission Proteins Regulate Programmed Cell Death in Yeast. Genes Dev..

[52] Arnoult D., Rismanchi N., Grodet A., Roberts R.G., Seeburg D.P., Estaquier J., Sheng M., Blackstone C. (2005). Bax/Bak-Dependent Release of DDP/TIMM8a Promotes Drp1-Mediated Mitochondrial Fission and Mitoptosis during Programmed Cell Death. Curr. Biol..

[53] Estaquier J., Arnoult D. (2007). Inhibiting Drp1-Mediated Mitochondrial Fission Selectively Prevents the Release of Cytochrome C during Apoptosis. Cell Death Differ..

[54] Cheng W.C., Teng X., Park H.K., Tucker C.M., Dunham M.J., Hardwick J.M. (2008). Fis1 Deficiency Selects for Compensatory Mutations Responsible for Cell Death and Growth Control Defects. Cell Death Differ..

[55] Cereghetti G.M., Stangherlin A., Martins de Brito O., Chang C.R., Blackstone C., Bernardi P., Scorrano L. (2008). Dephosphorylation by Calcineurin Regulates Translocation of Drp1 to Mitochondria. Proc. Natl. Acad. Sci. USA.

[56] Cribbs J.T., Strack S. (2007). Reversible Phosphorylation of Drp1 by Cyclic Amp-Dependent Protein Kinase and Calcineurin Regulates Mitochondrial Fission and Cell Death. EMBO Rep..

[57] Saotome M., Safiulina D., Szabadkai G., Das S., Fransson A., Aspenstrom P., Rizzuto R., Hajnoczky G. (2008). Bidirectional Ca^2+^-Dependent Control of Mitochondrial Dynamics by the Miro GTPase. Proc. Natl. Acad. Sci. USA.

[58] Slupe A.M., Merrill R.A., Flippo K.H., Lobas M.A., Houtman J.C., Strack S. (2013). A Calcineurin Docking Motif (LXVP) in Dynamin-Related Protein 1 Contributes to Mitochondrial Fragmentation and Ischemic Neuronal Injury. J. Biol. Chem..

[59] Strack S., Wilson T.J., Cribbs J.T. (2013). Cyclin-Dependent Kinases Regulate Splice-Specific Targeting of Dynamin-Related Protein 1 to Microtubules. J. Cell Biol..

[60] Qi X., Disatnik M.H., Shen N., Sobel R.A., Mochly-Rosen D. (2011). Aberrant Mitochondrial Fission in Neurons Induced by Protein Kinase C{delta} Under Oxidative Stress Conditions in Vivo. Mol. Biol. Cell.

[61] Hong Z., Kutty S., Toth P.T., Marsboom G., Hammel J.M., Chamberlain C., Ryan J.J., Zhang H.J., Sharp W.W., Morrow E. (2013). Role of Dynamin-Related Protein 1 (Drp1)-Mediated Mitochondrial Fission in Oxygen Sensing and Constriction of the Ductus Arteriosus. Circ. Res..

[62] Prieto J., Leon M., Ponsoda X., Sendra R., Bort R., Ferrer-Lorente R., Raya A., Lopez-Garcia C., Torres J. (2016). Early ERK1/2 Activation Promotes Drp1-Dependent Mitochondrial Fission Necessary for Cell Reprogramming. Nat. Commun..

[63] Zhang C.S., Lin S.C. (2016). AMPK Promotes Autophagy by Facilitating Mitochondrial Fission. Cell Metab..

[64] Toyama E.Q., Herzig S., Courchet J., Lewis T.L.J., Loson O.C., Hellberg K., Young N.P., Chen H., Polleux F., Chan D.C. (2016). Metabolism. AMP-Activated Protein Kinase Mediates Mitochondrial Fission in Response to Energy Stress. Science.

[65] Francy C.A., Alvarez F.J., Zhou L., Ramachandran R., Mears J.A. (2015). The Mechanoenzymatic Core of Dynamin-Related Protein 1 Comprises the Minimal Machinery Required for Membrane Constriction. J. Biol. Chem..

[66] Bustillo-Zabalbeitia I., Montessuit S., Raemy E., Basanez G., Terrones O., Martinou J.C. (2014). Specific Interaction with Cardiolipin Triggers Functional Activation of Dynamin-Related Protein 1. PLoS ONE.

[67] Stepanyants N., Macdonald P.J., Francy C.A., Mears J.A., Qi X., Ramachandran R. (2015). Cardiolipin’s Propensity for Phase Transition and Its Reorganization by Dynamin-Related Protein 1 form a Basis for Mitochondrial Membrane Fission. Mol. Biol. Cell.

[68] Clinton R.W., Francy C.A., Ramachandran R., Qi X., Mears J.A. (2016). Dynamin-Related Protein 1 Oligomerization in Solution Impairs Functional Interactions with Membrane-Anchored Mitochondrial Fission Factor. J. Biol. Chem..

[69] Adachi Y., Itoh K., Yamada T., Cerveny K.L., Suzuki T.L., Macdonald P., Frohman M.A., Ramachandran R., Iijima M., Sesaki H. (2016). Coincident Phosphatidic Acid Interaction Restrains Drp1 in Mitochondrial Division. Mol. Cell.

[70] Macdonald P.J., Stepanyants N., Mehrotra N., Mears J.A., Qi X., Sesaki H., Ramachandran R. (2014). A Dimeric Equilibrium Intermediate Nucleates Drp1 Reassembly on Mitochondrial Membranes for Fission. Mol. Biol. Cell.

[71] Frohlich C., Grabiger S., Schwefel D., Faelber K., Rosenbaum E., Mears J., Rocks O., Daumke O. (2013). Structural Insights into Oligomerization and Mitochondrial Remodelling of Dynamin 1-Like Protein. EMBO J..

[72] Koirala S., Guo Q., Kalia R., Bui H.T., Eckert D.M., Frost A., Shaw J.M. (2013). Interchangeable Adaptors Regulate Mitochondrial Dynamin Assembly for Membrane Scission. Proc. Natl. Acad. Sci. USA.

[73] Song W., Chen J., Petrilli A., Liot G., Klinglmayr E., Zhou Y., Poquiz P., Tjong J., Pouladi M.A., Hayden M.R. (2011). Mutant Huntingtin Binds the Mitochondrial Fission Gtpase Dynamin-Related Protein-1 and Increases Its Enzymatic Activity. Nat. Med..

[74] Yoon Y., Pitts K.R., Mcniven M.A. (2001). Mammalian Dynamin-Like Protein DLP1 Tubulates Membranes. Mol. Biol. Cell.

[75] Macdonald P.J., Francy C.A., Stepanyants N., Lehman L., Baglio A., Mears J.A., Qi X., Ramachandran R. (2016). Distinct Splice Variants of Dynamin-Related Protein 1 Differentially Utilize Mitochondrial Fission Factor as an Effector of Cooperative GTPase Activity. J. Biol. Chem..

[76] Francy C.A., Clinton R.W., Fröhlich C., Murphy C., Mears J.A. (2017). Cryo-EM Studies of Drp1 Reveal Cardiolipin Interactions That Activate the Helical Oligomer. Sci. Rep..

[77] De Brito O.M., Scorrano L. (2008). Mitofusin 2 Tethers Endoplasmic Reticulum to Mitochondria. Nature.

[78] Hayashi T., Rizzuto R., Hajnoczky G., Su T.P. (2009). Mam: More than Just a Housekeeper. Trends Cell Biol..

[79] Mironov S.L., Symonchuk N. (2006). ER Vesicles and Mitochondria Move and Communicate at Synapses. J. Cell Sci..

[80] Shiao Y.J., Lupo G., Vance J.E. (1995). Evidence That Phosphatidylserine is Imported into Mitochondria via a Mitochondria-Associated Membrane and That the Majority of Mitochondrial Phosphatidylethanolamine is Derived from Decarboxylation of Phosphatidylserine. J. Biol. Chem..

[81] Friedman J.R., Lackner L.L., West M., Dibenedetto J.R., Nunnari J., Voeltz G.K. (2011). ER Tubules Mark Sites of Mitochondrial Division. Science.

[82] Korobova F., Ramabhadran V., Higgs H.N. (2013). An Actin-Dependent Step in Mitochondrial Fission Mediated by the ER-Associated Formin INF2. Science.

[83] Kornmann B., Currie E., Collins S.R., Schuldiner M., Nunnari J., Weissman J.S., Walter P. (2009). An ER-Mitochondria Tethering Complex Revealed by a Synthetic Biology Screen. Science.

[84] Toulmay A., Prinz W.A. (2012). A Conserved Membrane-Binding Domain Targets Proteins to Organelle Contact Sites. J. Cell Sci..

[85] Voss C., Lahiri S., Young B.P., Loewen C.J., Prinz W.A. (2012). ER-Shaping Proteins Facilitate Lipid Exchange between the ER and Mitochondria in *S. cerevisiae*. J. Cell Sci..

[86] Murley A., Lackner L.L., Osman C., West M., Voeltz G.K., Walter P., Nunnari J. (2013). ER-Associated Mitochondrial Division Links the Distribution of Mitochondria and Mitochondrial DNA in Yeast. eLife.

[87] Chhabra E.S., Higgs H.N. (2006). INF2 is a WASP Homology 2 Motif-Containing Formin That Severs Actin Filaments and Accelerates both Polymerization and Depolymerization. J. Biol. Chem..

[88] Lee J.E., Westrate L.M., Wu H., Page C., Voeltz G.K. (2016). Multiple Dynamin Family Members Collaborate to Drive Mitochondrial Division. Nature.

